# Supplementary material for: Influence of vitamin E and carcass feeding supplementation on fecal glucocorticoid and androgen metabolites in male black-footed ferrets (Mustela nigripes)
Source: PLoS One. 2020 Oct 23;15(10):e0241085. doi: 10.1371/journal.pone.0241085 (PMC7584211; doi:10.1371/journal.pone.0241085)
Supplement: Attachment — Submitted filename: review for PONE-D-20-09363.docx [file pone.0241085.s002.docx]

**Abstract**

Line 32: enzyme immunoassay (no “s”)

Line 39: Remove the extra “the”; explicitly state that age was not significant so the reader is not looking for those data in the lines that follow

Line 41: Does “supplemented” refer to vitamin E or carcass? The abstract needs to be more consistent throughout so that it is clear what “supplemented” is referring to in each sentence.

**Introduction**

Line 63: No need to repeat “muscle”

Line 63-64: The authors have already stated it is difficult to replicate.

Line 75: Suggest “improving physical health” (vs. dental alone)

Line 78: Do the authors mean “psychological” here (since physiological was discussed in the previous paragraph and the current paragraph seems to focus on behavior)?

Line 102: normal sperm acrosome

Line 106: Please quantify “nearly coincides”

Line 119-122: The authors did not really examine stress or psychological enrichment. They examined *adrenal* and reproductive biomarkers, and the first hypothesis should be revised to state simply that FGM would be lower in ferrets fed whole prey items.

Overall, this section is well-written, interesting, and presents good reason for conducting the study.

**Methods**

Lines 135-137: Perhaps this can be combined with the sentences at the start of this section to consolidate?

Line 138: Sample size was stated in the previous paragraph but with less detail regarding sample sizes of the age groups. Consolidate all this information in the same sentence.

Line 143: Presumably, “adult” is the 2 and 3 year-olds? The authors have not yet talked about the samples, so it might be clearer to state simply “a subset of adult males…was used to evaluate…” Lastly, there are 4 treatment groups, but this subset in which FAM will be measured appears to be divided into only 2, yet is to be used for evaluating effects of vit. E **and/or** carcass. If it is for evaluating vit. E plus either carcass or no carcass, perhaps in that case the authors can state, “a subset of adult males in groups 2 and 3 was used to evaluate…”. Otherwise, there would seem to be two missing groups from this subset (no vit E + carcass; no vit E no carcass)? Indeed, the results suggest the comparison of importance is between vitamin E versus no vitamin E, so is that really group 3 vs. group 4 were vitamin E supplementation differs but both receive carcass?

Line 149: Were all these samples collected at the same time of day or, if not, have previous publications already demonstrated that the circadian rhythm in glucocorticoid concentrations does not appear in fecal samples collected at different times of day (i.e., the excretory lag time is sufficiently long to “average out” the daily variation in FGM concentrations)? If not collected at the same time of day and if not previously demonstrated that FGM do not vary across the day, perhaps this accounts for some of the important but unexplained variation in the statistical models mentioned in the discussion?

Line 150: It is unfortunate there could not be a breeding season, no diet change combination.

Line 151: Samples were stored at **minus** 20C

Line 154: were dried

Line 158: It would be clearer to state the residual fecal material was resuspended rather than the “samples” to avoid confusion with the first supernatant that was decanted.

Line 161: 0.5 ml

Line 165: immunoassay

Line 167: Remove extra “(“

Line 171, 172; Line 180, 181: Please provide standard values in pg/ml (or similar units) or provide assay details of how many µl are added per well so the reader is able to do the necessary calculations if they wish to repeat the methods. Also provide assay sensitivity in pg/ml or similar.

Line 174: Sample size already described earlier in this section

Line 174-175: analyzed by or analyzed using

Line 187-188: It was described earlier that juveniles were not sampled for the FAM analysis, only adults, so presumably this statement is irrelevant?

Clarification is needed in this section with respect to the experimental design for the FAM analysis. Otherwise, only minor edits to clarify details are needed.

**Results**

Line 222: Suggest “from pre-breeding season to breeding season time periods”

Line 220-231: I think these data will be more easily understood by the audience if the authors take us through the “story” told by these data chronologically. Currently, it describes what happens to each group of males (carcass-supplemented or not) separately, then compares them within the seasons, and we have to go back and remember what the differential responses were across those seasons. It could be better to say, “in pre-breeding season, males that would be fed carcass in the coming months had X FGM concentrations compared to those that would not be supplemented. Once carcass was fed, the males receiving it experienced X whereas those not receiving it experienced Y, and these concentrations differed from each other. By post-breeding season…” I think this will better communicate the message about FGM differences to the audience than the way it is currently presented.

Line 232: Suggest stating p = 0.07 rather than p < 0.10

Line 232-243: Again, I suggest laying these data out chronologically to better communicate the differences. Also, from Table 2, it looks like carcass had no effect on FAM and did not interact with vitamin E, but Figure 2 suggests there was no carcass supplementation going on at all. I suspect Figure 2 should state that treatment was “(carcass + vitamin E vs. carcass with no vitamin E)”. I was unclear about the break-down of the FAM groups in the methods as well. In addition to clarifying this in the methods, it would be helpful to state explicitly in the results that the described results for vit. E on FAM occurred for those on carcass in the same manner as they occurred for those not on carcass (or were only assessed for those on carcass?).

Overall, the data are all here, but the way they are organized leaves the reader to try and piece together a complete picture from the components, which I fear could result in losing the point the authors will want to make. If these paragraphs could be re-written to better focus the reader on the critical differences at the critical points in time, the authors will have the readers “on board” by the time we get to the discussion. The figures are quite nice and allow the audience to interpret the data (except it is still unclear if the vitamin E data are for animals that were on carcass, not on carcass, or both).

**Discussion**

Line 264-282: These previous findings provide the initial rational for testing supplemental carcass feeding, but perhaps this could be more briefly summarized since it was already mentioned in the introduction as well? The study itself does not generate any dental data, yet the topic kicks-off and dominates the first 20 lines of the discussion.

Line 282-288: While interesting, this deals with skeletal changes as a result of space/activity, which has nothing to do with the present study.

Line 288-293: Again, while that might be interesting to consider, none of the data presented in this study speaks to morphological changes, so this musing seems unnecessary.

Line 294-297: It could be reasonable that whole carcass is more psychologically enriching, but no behavioral data were presented in this study. There is no pre-carcass behavioral data (or non-FGM physiological data that could be used as an indicator of stress) to support the notion that the ferrets were stressed to begin with, nor were any behavioral (or other) data recorded to support the notion that stress was reduced by carcass feeding. The authors acknowledge the absence of this data in subsequent lines and suggest instances in other studies where behavior was improved by carcass feeding, but the suggested conclusion in ferrets is entirely based on supposition.

Glucocorticoids are metabolic hormones, and while the psychological stress response can require a change in metabolism, metabolic change associated with breeding condition and associated with a change in diet/digestion (both of which are featured in this study) can occur in the absence of a change in psychological state. The authors go too far in concluding that their data suggest a reduction in stress when the only parameter measured is glucocorticoid metabolites. The authors have demonstrated a change in metabolism, and as it was in the direction opposite of those ferrets that did not receive carcass during the breeding season, the change does indeed appear to be a result of the carcass supplementation, but the effects of that supplementation are unknown at this point in terms of why it caused a change in metabolism and what it means for the ferrets. Instead of a change in psychological stress, couldn’t FGM have been reduced because there was more bone and fibrous tissue to attempt to digest and less protein and simple carbohydrate? It would be very helpful to have weight data on these different groups to see what effect the diet change had on their weight gain/loss. Perhaps this information is available in the ZIMS records and can be analyzed to provide some insight here? Another option to consider if the authors feel strongly that the change in FGM is indeed related to stress is to test another biomarker for stress. Markers of oxidative stress have been measured using fecal samples in other species—possibly try Arbor Assays K059?

Line 311-313: If improved oral health is tied to carcass supplementation, and carcass supplementation continued in the post-breeding season, then shouldn’t FGM continue to be lower in the carcass group than the non-carcass group in the post-breeding season? Instead, they are the same. This suggests that the effects of carcass supplementation on FGM concentrations were strongest or most relevant to the biology of the ferret during breeding season in particular. It would seem that a relationship to oral health should be fairly consistent across the year, no? The authors do bring up the interesting idea of glucocorticoid-mediated suppression of the immune system in subsequent lines. I wonder if perhaps there is literature on how the immune response of ferrets during the breeding season is up- or down-regulated normally and if that could then have any relationship to the differential FGM response of the ferrets in this study?

Line 323: What is the situation in the managed population? Are males and females paired individually according to genetics or is there some opportunity for competition/choice?

Line 336: Is it possible to add the no-effect-of-carcass data to the FAM graph? It is interesting that although FGM increased in the non-carcass group whereas FGM decreased in the carcass group, the carcass group did not manage to have higher FAM (since FGM might suppress FAM). Perhaps FAM were higher in the carcass group, it just was not statistically significant, especially since the authors noted an improvement in semen characteristics in a previous study of carcass supplementation?

Line 328-347: Within this paragraph, the connection between the fact that FAM increased more during the breeding season in the ferrets that received vitamin E supplementation than those that did not (line 335-336) and the mechanism for that relationship and beneficial effects related to semen (lines 343-347) gets lost by the intervening information about the lack of relationships in this study between carcass and FAM and between vitamin E and FGM. The important vitamin E-FAM relationship probably needs to be in its own paragraph, while the absent carcass-FAM and vitamin E-FGM relationships can be in a separate paragraph. Your topic sentences for the paragraph beginning on line 367 could be topic sentences for these paragraphs, especially since they are not related to the subsequent lines of the paragraph in which they currently appear (370-375).

Line 360-364: It sounds like the issue might be an organizational development issue. Have you considered estrogens in the TOR diet or in the water source?

Line 369-373: Perhaps all this variation that is important but unexplained by the fixed effects also suggests, along with your fecundity data, that some other fixed effect must be tested in order to solve this ferret fertility issue.

Line 379-380: Again, I really do not think the authors can point to what about carcass feeding contributed to the lower FGM in carcass-fed ferrets with this data set and with the discussion points that are presented. If attributed to improved dental health or enriching qualities, I would think those effects should have continued into the post-breeding season. The authors should consider what could be special about the physiology and metabolism of the ferrets during the breeding season in particular that could better explain this interesting and possibly important finding.

The discussion brings up a lot of interesting points, but the authors need to discuss the data in this study a bit more thoroughly. There seems to be something missing from how the authors are thinking about the change in FGM that could be better explored. Possibly more data needs to be gathered (weight, other biomarkers of stress, even behavioral notes from keepers), and at this time the most that can be said is that a change occurred but additional study is necessary to understand why and to understand its potential effects. The authors should be very careful about concluding that there was any change in psychological stress—it is possible but remains unsupported at this time. The part of the discussion related to FAM could be better organized to draw attention to the essential finding(s) and how that relates to previous studies but does not fully solve the ferret reproduction issue.

The study on the whole is interesting and important, but clarification is necessary in the experimental design, and a more focused interpretation of the data presented by this study so that it neither over-states nor under-states possible conclusions is needed. Alternative interpretations should be sought for the FGM data as those provided in the discussion do not seem to adequately explain the findings.
