# Supplementary material for: Influence of vitamin E and carcass feeding supplementation on fecal glucocorticoid and androgen metabolites in male black-footed ferrets (Mustela nigripes)
Source: PLoS One. 2020 Oct 23;15(10):e0241085. doi: 10.1371/journal.pone.0241085 (PMC7584211; doi:10.1371/journal.pone.0241085)
Supplement: Attachment — Submitted filename: review for PONE-D-20-09363_R1.docx [file pone.0241085.s004.docx]

**The paper should be accepted, however I do have a few suggested edits that I think are important.**

**Introduction**

Line 112: So, the problems with reproduction, at least from the perspective of semen quality, pre-dated the diet change, i.e., were unrelated to the diet change? If so, while it is still very important to get the diet as good as it can be for numerous reasons, this timing suggests the specific change made in 2001 is not the culprit for reproductive decline (though perhaps it is not helping the situation either).

Line 115: Missing the “)” after “Canada”

**Methods**

Lines 137: Seems to be something missing from the second half of this sentence

Lines 148-152: Groups are much clearer now—thank you

Line 163-164: The way it is currently presented with the parenthetical notes, it still seems like the control group was not sampled February-June, but I see your reply in the reviewer notes. I suggest stating that samples were collected from all 4 groups at each time point but no diet changes were initiated in treatment groups until February.

**Results**

Much clearer—thank you.

**Discussion**

Line 301: Remove “is largely”

Line 301-302: Suggested revision for clarity: “However, the carcass supplementation did lower FGM enough during breeding season that is was not significantly different than post-breeding season.”

Line 363-364: Intro states 60% to 35%

Line 376: Again, because the decline in reproduction was noted a year before the diet change, stating that “diet may be the variable that is leading to infertility” could be a bit strong. It might not help, to be sure, but I agree a more detailed study is required.

Line 401: I believe the authors mean to say “because ***glucocorticoids*** can suppress the immune response” based on the rest of the sentence discussing carcass addition and its potential benefits.

This section is better organized, the take-home-messages more clearly stated, and the statements better supported than in my first review. Alternative explanations are appropriately noted. Just a thought: If vit E supplementation begins in pre-breeding season as males are preparing for increased spermatogenesis (v. starting after the fact during breeding season), perhaps there might be a better outcome for reproduction…
