# Supplementary material for: Influence of vitamin E and carcass feeding supplementation on fecal glucocorticoid and androgen metabolites in male black-footed ferrets (Mustela nigripes)
Source: PLoS One. 2020 Oct 23;15(10):e0241085. doi: 10.1371/journal.pone.0241085 (PMC7584211; doi:10.1371/journal.pone.0241085)
Supplement: Attachment — Submitted filename: Response to reviewers revision I 100520.docx [file pone.0241085.s005.docx]

**Thank you for your time and effort. You have helped to clarify and strengthen our manuscript. Below in bold are our responses.**

Reviewer #1: Introduction
Line 112: So, the problems with reproduction, at least from the perspective of semen quality, pre-dated the diet change, i.e., were unrelated to the diet change? If so, while it is still very important to get the diet as good as it can be for numerous reasons, this timing suggests the specific change made in 2001 is not the culprit for reproductive decline (though perhaps it is not helping the situation either).
**Yes, the males collected from in 2001 were for artificial insemination program because they weren’t siring on their own and were genetically valuable. This might have been due to poor semen quality. We have removed this sentence in the introduction.**

Line 115: Missing the “)” after “Canada”

**Corrected**

Methods
Lines 137: Seems to be something missing from the second half of this sentence

**Corrected**

Lines 148-152: Groups are much clearer now—thank you

**You are welcome. Thank you for the helpful comments.**

Line 163-164: The way it is currently presented with the parenthetical notes, it still seems like the control group was not sampled February-June, but I see your reply in the reviewer notes. I suggest stating that samples were collected from all 4 groups at each time point but no diet changes were initiated in treatment groups until February.

**We have clarified sample collection and when diet change occurred.**

Results
Much clearer—thank you.

**You are welcome. Thank you for the helpful comments.**

Discussion
Line 301: Remove “is largely”

**Corrected**

Line 301-302: Suggested revision for clarity: “However, the carcass supplementation did lower FGM enough during breeding season that is was not significantly different than post-breeding season.”

**Corrected**

Line 363-364: Intro states 60% to 35%

**That was since 2000. Originally, whelping rates were around 80%. We clarified the sentence to make that point clear.**

Line 376: Again, because the decline in reproduction was noted a year before the diet change, stating that “diet may be the variable that is leading to infertility” could be a bit strong. It might not help, to be sure, but I agree a more detailed study is required.

**Hopefully, with the clarification about the males 2001 being AI males because they were not siring on their own, demonstrates why we are investigating diet further. With this several generations diet study, we hope to show results that suggest more than correlation, but causation (if possible without laboratory experimentation).**

Line 401: I believe the authors mean to say “because glucocorticoids can suppress the immune response” based on the rest of the sentence discussing carcass addition and its potential benefits.

**We did mean “testosterone”. We stated this because GCs went down during the breeding season but testosterone increased with the addition of carcass. The increased testosterone did not seem to improve reproductive success. So our point was that high testosterone has its costs, but the carcass might help to mitigate them along with providing enrichment/a distraction. But, we removed it and kept the conclusions more general.**

This section is better organized, the take-home-messages more clearly stated, and the statements better supported than in my first review. Alternative explanations are appropriately noted. Just a thought: If vit E supplementation begins in pre-breeding season as males are preparing for increased spermatogenesis (v. starting after the fact during breeding season), perhaps there might be a better outcome for reproduction…

**Thanks! We hope our 3 year diet change study will address this thought/idea.**

Reviewer #2: Thank you for the changes that were made. Overall, it reads much better. However, I am still struggling with the Introduction. There are few reasons a paper should have a 3 1/2 page introduction. The first 3 paragraphs do not outline the problem at all but instead discuss nutrition, diet formulation and dental health. The gist of the paper's background very nicely starts on Line 88 with a very nice presentation of the history and issues. That then follows very nicely with the need to evaluate vitamin E starting on line 122. It seems that right after Line 128 you could add a bit about the behavior impact/stress on reproduction (starting from Line 71) then introduce the concept of whole prey. That would allow your ending objective statement to flow much better. Regardless the first 2 paragraphs do not benefit your paper in any way and take away from the actual problem that you don't start talking about until Line Line 88. This is about trying to enhance the diets for the BFF program not formulate diets and discuss nutrition for all zoo managed carnivores.

**We have greatly reduced the first three paragraphs in the introduction. We made the suggested organizational changes, but kept a couple of sentences, such as the lead into nutrition’s importance to welfare, difficulty of specialized carnivore diets and the how the addition of prey items may reduce stress.**  **We wanted some of the literature to set up the difficulties of feeding BFF, which is a specialized carnivore, and why adding carcass could affect GCs (through behavioral modifications).**

Line 67: What is "processed"? Do you mean basically a TOR that you define later (line 115)? A definition of processed should be included...is that simply ground meat that is complete? I think you mean TOR but you should define it sooner if you are referring to it here.

**Based on your suggested intro re-organization, this sentence has been deleted.**

Line 141 should read Animals and Diet since that section is not just about the animals or it could be Animals and Treatments

**Corrected**

Line 150: Add "diet" between Kg and Dry Matter Basis
**Corrected**

Line 150 - 152: It is unclear if treatments 3 and 4 have the same whole/prey composition. Is "two hamsters or prairie dogs
pieces/week (3) the same thing as carcass item twice weekly (4)? Was there any attempt made to control/standardize the weight of carcass items for these 2 treatments. As it's currently written it doesn't appear to be the same.

**Yes, it was the same. And, yes, weight of the carcass item was standardized. We clarified this in the text.**
